# Cloning, characterization, and heterologous expression of a candidate Hirudin gene from the salivary gland transcriptome of *Hirudo nipponia*

**DOI:** 10.1038/s41598-023-32303-2

**Published:** 2023-03-27

**Authors:** Ping Shi, Jian Wei, Huajian You, Shijiang Chen, Fayin Tan, Zenghui Lu

**Affiliations:** 1 Institute of Chinese Caterpillar Fungus, Chongqing Academy of Chinese Materia Medica, Chongqing, 400065 People’s Republic of China; 2 Chongqing College of Traditional Chinese Medicine, Chongqing, 402760 People’s Republic of China; 3 Chongqing Sub-Center of National Resource Center for Chinese Materia Medica, China Academy of Chinese Medical Science, Chongqing, 400065 People’s Republic of China; 4 Department of TCM Geriatrics, Pucheng County Hospital, Shaanxi, 715500 People’s Republic of China

**Keywords:** Genetic engineering, Synthetic biology

## Abstract

Hirudin is a pharmacologically active substance in leeches with potent blood anticoagulation properties. Although recombinant hirudin production isolated from *Hirudo medicinalis* Linnaeus and *Hirudinaria manillensis* Lesson is known, to our knowledge, this study is the first to report recombinant hirudin expression and production from *Hirudo nipponia* Whitman. Thus, the present study aimed to clone and characterize the full-length cDNA of a candidate hirudin gene (c16237_g1), which is localized on the salivary gland transcriptome of *H. nipponia*, and further evaluate its recombinant production using a eukaryotic expression system. The 489-bp cDNA possessed several properties of the hirudin “core” motifs associated with binding to the thrombin catalytic pocket. A fusion expression vector (pPIC9K-hirudin) was constructed and successfully transformed into *Pichia pastoris* strain GS115 via electroporation. Sodium dodecyl sulphate–polyacrylamide gel electrophoresis and western blot analysis confirmed hirudin expression. The recombinant protein was expressed with a yield of 6.68 mg/L culture. Mass spectrometry analysis further confirmed target protein expression. The concentration and antithrombin activity of purified hirudin were 1.67 mg/mL and 14,000 ATU/mL, respectively. These findings provide a basis for further elucidating the molecular anticoagulation mechanism of hirudin, and address China’s growing market demand for engineered *H. nipponia*-derived hirudin and hirudin-based drugs.

## Introduction

*Hirudo nipponia* is a blood-sucking leech that has been reported to possess substantial medicinal value in traditional Chinese medicine, which is first recorded in the classic book on Chinese Materia Medica, *Shen-Nong-Ben-Cao-Jing* (ca. 100 AD)^1–3^. *H. nipponia* has been widely used to treat cardiovascular and cerebrovascular diseases, as well as cerebral thrombosis, coronary heart disease, and cerebral edema^4–6^. It is also listed in the Pharmacopoeia of the People’s Republic of China owing to its powerful antithrombin activity^7^.

One of the main pharmacologically active substances in blood-sucking leeches is hirudin, which is a naturally occurring peptide with blood anticoagulant property, produced in the salivary glands of *H. nipponia*. It is the strongest natural thrombin-specific inhibitor identified to date^8–11^. Hirudin and several hirudin analogues possess a specific anticoagulant effect and are widely used in clinical settings. Among these, two recombinant hirudins and a hirudin analogue have gained marketing approval from the United States Food and Drug Administration, for various applications^12^. The anticoagulant activity of *H. nipponia* is significantly higher than those of the other two leech species (*Whitmania pigra* Whitman and *Whitmania acranulata* Whitman) listed in the Pharmacopoeia of the People’s Republic of China^13^.

Wild *H. nipponia* species have experienced a sharp population decline due to environmental pollution, over-harvesting, and large-scale habitat destruction, resulting in the depletion of raw material resources to meet the high demand for hirudin^13,14^. Despite progress in artificial breeding, the commercial production of this medicinal leech is yet to be realized^13^. Thus, safe and effective methods to produce hirudin are needed to prevent endangering natural wild *H. nipponia*, thereby preventing the depletion of natural resources while simultaneously promoting the modernization of traditional Chinese medicine. Hence, utilizing genetically engineered microorganisms to produce recombinant hirudin is a potentially effective strategy^15–17^.

Natural hirudin exists in three isoforms (HV1, HV2, and HV3), comprising 65 or 66 amino acid residues, with a molecular weight of approximately 7000 Da^18–22^. Recently, genetic recombinant technology has been used to meet pharmacological and clinical demands through bacterial and eukaryotic expression systems, including *Escherichia coli* and *Pichia pastoris*, which secrete recombinant hirudin isolated from *H. medicinalis* and *Hirudinaria manillensis* Lesson^15–18,23–25^. However, few studies have focused on expressing the hirudin gene and the production of recombinant hirudin from *H. nipponia* using molecular biotechnology techniques^26,27^.

In a previous study^27^, we showed that a candidate hirudin gene (c16237_g1) is localized on the salivary gland transcriptome of *H. nipponia.* Accordingly, the present study aimed to clone and characterize the full-length cDNA sequence of c16237_g1*.* We further aimed to evaluate the use of a eukaryotic expression system for recombinant hirudin production. To the best of our knowledge, this is the first report of expressing recombinant hirudin protein from *H. nipponia*. Therefore, this study will lay the foundation for further research in determining the molecular anticoagulation mechanism of hirudin. It will also address China’s current market requirement for engineered *H. nipponia* hirudin and meet the huge demand for hirudin-based clinical drugs, including products targeting important cardiovascular and cerebrovascular diseases.

## Results

### Molecular cloning of the hirudin gene in *H. nipponia*

**Figure 1:**
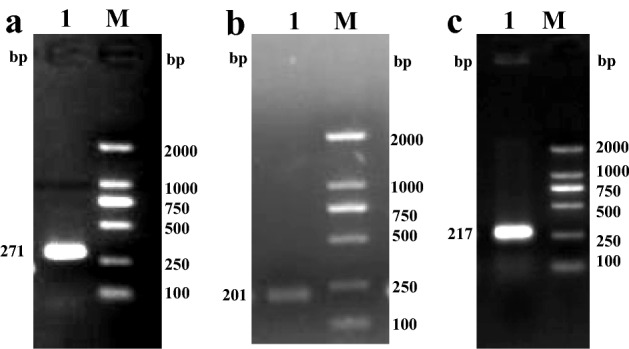
Amplification of a candidate hirudin gene from the salivary gland transcriptome of *Hirudo nipponia*. (**a**) Intermediate fragment amplification. (**b**) 5′-rapid amplification of cDNA ends (RACE) amplification. (**c**) 3′-RACE amplification (1: Target fragment; M: Marker DL2000). The polymerase chain reaction (PCR) products were analyzed on a 1% agarose gel. The original gel is presented in Fig. S1.

**Figure 2:**
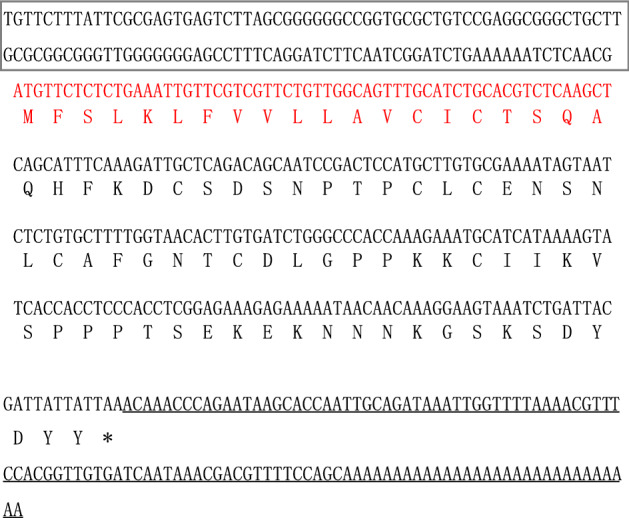
Full-length cDNA sequence of hirudin. (The signal peptide is marked in red; the initiation codon is shown in bold; the termination codon is indicated by an asterisk; 5′-terminal untranslated region (UTR*)* is shown in box; 3′-terminal UTR is marked with a straight line; and the other parts represent the mature hirudin amino acid sequence).

Based on the annotated hirudin transcript (c16237_g1) (sequence shown in Supplementary data) in the salivary gland transcriptome data of *H. nipponia*, one intermediate fragment of 271 bp, a 201-bp fragment amplified using 5′-RACE, and a 217-bp fragment using 3′-RACE (Fig. 1) were assembled using DNASTAR ver. 7.1 to obtain the full-length sequence of the hirudin gene in *H. nipponia*. The full-length cDNA of hirudin was 489 bp, including a 5′-terminal untranslated region (UTR) of 123 bp, a 3′-terminal UTR of 114 bp, and a 252-bp open reading frame (ORF) encoding an 83-aa protein polypeptide. The nucleotide and predicted amino acid sequences of the full-length cDNA are shown in Fig. 2. The complete hirudin cDNA sequence has been deposited in the GenBank database under accession number GenBank MN 116511.

### Sequence analysis and multiple sequence alignment of hirudin

The 489-bp full-length cDNA sequence encoded an 83-aa hirudin protein, containing a 20-aa N-terminal signal peptide and 63-aa mature protein sequence (Fig. 2). After removing the signal peptide, the predicted molecular weight and the theoretical isoelectric point (pI) of the mature protein were 6.97 kDa and 6.72, respectively. The protein contained eight negatively charged residues (Asp and Glu) and eight positively charged residues (Arg and Lys). The aliphatic index was 37.14 and the grand average of hydropathicity was − 1.070.

**Figure 3:**
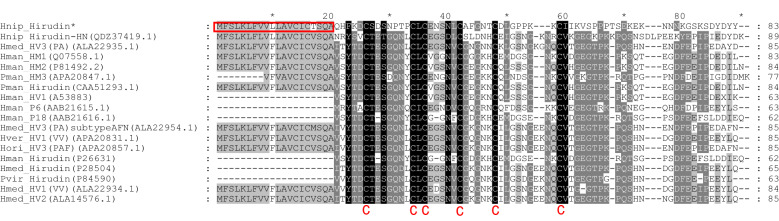
Alignment of hirudin protein amino acid sequences (*: candidate sequence from the present study; red box: predicted secretory signal peptides; *C* conserved cysteines residues, *Hnip* *Hirudo nipponia*, *Hmed* *H. medicinalis*, *Hman* *Hirudinaria manillensis*, *Pman* *Poecilobdella manillensis*, *Hver* *H. verbena*, *Hori* *H. orientalis*, *Pvir* *Poecilobdella viridis*).

Homologous protein search using the National Center for Biotechnology Information (NCBI)-blastp tool in GenBank revealed 62, 60, 54, 53, 53, 53, 52, and 48% similarities to the *H. medicinalis* hirudin variant HV3 (PA) (ALA22935.1), *H. manillensis* hirudin variant HV1 (P81492.2), *H. medicinalis* hirudin variant HV1 (VV) (ALA22934.1), *H. medicinalis* hirudin variant HV2 (ALA14576.1), *H. verbana* hirudin variant HV1-VV (APA20831.1), *H. orientalis* hirudin variant HV3-PAF (APA20857.1), *H. manillensis* hirudin-HM1 (Q07558.1), and *Poecilobdella manillensis* hirudin (CAA51293.1), respectively. The multiple sequence alignment of hirudin is shown in Fig. 3, in accordance with the known structural information in the hirudin database^26–28^. The highly conserved motif was ^34^CLC^36^; the GSNV region was conservatively replaced by the chemically similar ^38^NSNL^41^ sequence in *H. nipponia*; and six cysteines residues, presumably involved in three disulfide bonds, were also found to be evolutionarily conserved.

### Restriction analysis of the recombinant plasmid pPIC9K-hirudin

**Figure 4:**
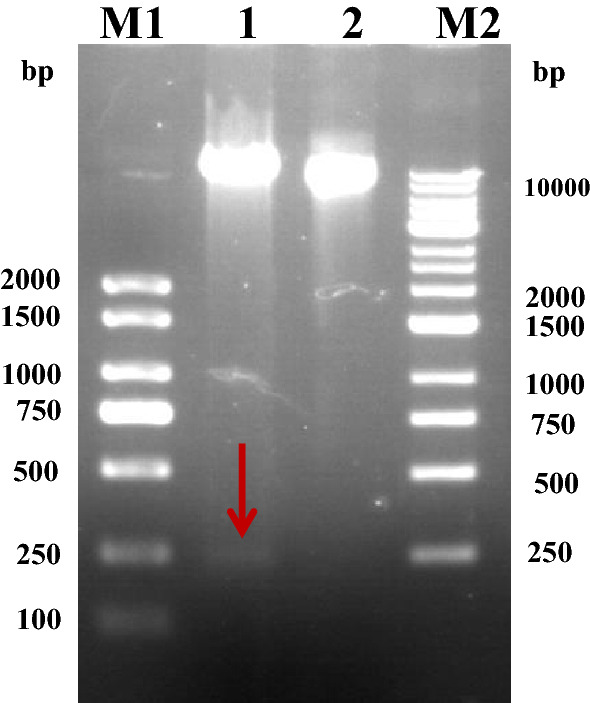
Analysis of the recombinant plasmid pPIC9K-hirudin through double enzyme digestion (Lane M1; Marker 2000; Lane 1: recombinant plasmid digested with EcoRI and NotI; Lane 2: recombinant plasmid; Lane M2: Marker DL 10,000). The target gene is indicated using a red arrow (217 bp). The products were analyzed on a 1% agarose gel. The original gel is presented in Fig. S2.

The positive transformants were screened and digested using EcoRI and NotI. Following double enzyme digestion, two bands were observed because the recombinant plasmid was 9276 bp in length and the target gene was approximately 217 bp in length. These results indicated that the recombinant plasmid pPIC9K-hirudin had been constructed successfully (Fig. 4).

### Polymerase chain reaction detection of the recombinant yeast transformant GS115/pPIC9K-hirudin

**Figure 5:**
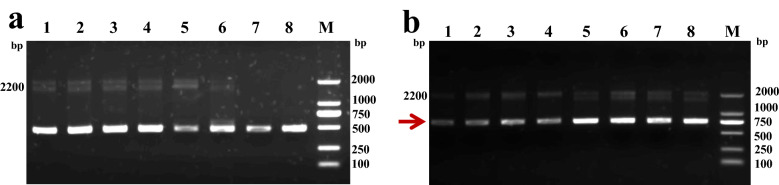
PCR detection of *Pichia pastoris* GS115 transformation with the recombinant plasmid pPIC9K-*Hirudin*. (**a**) *P. pastoris* GS115 cells transformed with the empty pPIC9K plasmid (GS115/pPIC9K). (**b**) *P. pastoris* GS115 transformants with pPIC9K-hirudin (GS115/pPIC9K-hirudin) (M: Marker DL2000). The recombinant integrated target gene is indicated using a red arrow (~ 720 bp). The PCR products were analyzed on a 1.5% agarose gel. The original gel is presented in Fig. S3.

Primers targeting the yeast alcohol oxidase 1 (*AOX1*) gene were used for polymerase chain reaction (PCR) amplification. Figure 5 shows two bands corresponding to sequences that were completely amplified in *P. pastoris* GS115/pPIC9K and GS115/pPIC9K-hirudin. A band of approximately the same size (~ 2200 bp) was identified as the *P. pastoris* GS115 *AOX1* gene^29,30^ along with a copy of the recombinant integrated target gene of approximately 720 bp (Fig. 5b). Thus, the PCR and sequencing results (shown in Supplementary sequence S2) demonstrated successful transformation of the recombinant plasmid pPIC9K-hirudin into *P. pastoris* GS115 cells.

### Target protein quantification and purification

**Figure 6:**
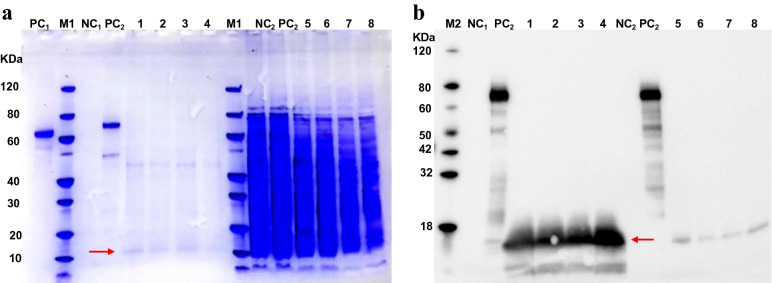
Sodium dodecyl sulphate–polyacrylamide gel electrophoresis (SDS-PAGE) and western blotting results of the induced products. (**a**) SDS-PAGE analysis of pPIC9K-hirudin expression in recombinant *Pichia pastoris*. (**b**) Western blot analysis of recombinant hirudin. (Lane M1: Protein MW marker (Broad); Lane M2: protein Marker (GenScript, Cat. No. M00673); Lane PC_1_: bovine serum albumin (1 µg); Lane PC_2_: positive control (GenScript, Cat. No. M0101); Lane NC_1_: medium without induction; Lane NC_2_: cell pellet without induction. Lane 1: supernatants from GS115/pPIC9K-hirudin after 24 h induction; Lane 2: Supernatants from GS115/pPIC9K-hirudin after 48 h induction; Lanes 3, 4: Supernatants from GS115/pPIC9K-hirudin after 72 h induction; Lane 5: Precipitate from GS115/pPIC9K-hirudin after 24 h induction; Lane 6: Precipitate from GS115/pPIC9K-hirudin after 48 h induction; Lanes 7, 8: Precipitate from GS115/pPIC9K-hirudin after 72 h induction.) The target protein is indicated using a red arrow (~ 15 kDa). Recombinant hirudin was analyzed on a 15% SDS-PAGE gel. The original gel is presented in Fig. S4.

**Figure 7:**
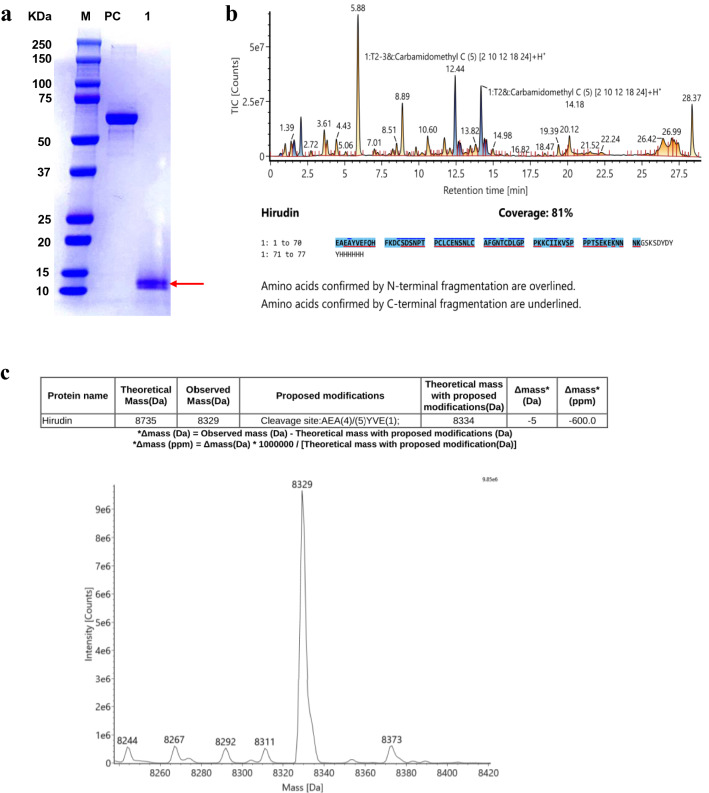
SDS-PAGE and mass spectrometric analysis of the purified protein. (**a**) SDS-PAGE detection of purified hirudin (M: Protein marker (Bio-rad, Cat. No. 1610374S). PC: BSA (2 µg). Lane 1: Purified hirudin.) (**b**) Tryptic digestion peptides and sequence of hirudin. (**c**) Liquid chromatography-tandem mass spectrometry detection of hirudin. The peak represents a mass of 8329 Da. Purified hirudin was analyzed on a 15% SDS-PAGE gel. The original gel is presented in Fig. S5.

Samples were analyzed via sodium dodecyl sulphate–polyacrylamide gel electrophoresis (SDS-PAGE) and western blotting after 24, 48, and 72 h induction. SDS-PAGE analysis, revealed a product purity of 95%. As indicated in Fig. 6, specific bands were visualized at approximately 15 kDa, thereby revealing successful target protein expression in the yeast culture supernatants. After expanded culture, induction, and purification, hirudin was successfully obtained in the supernatant, as determined using 15% SDS-PAGE (Fig. 7a). Liquid chromatography-tandem mass spectrometry (LC–MS/MS) results revealed the precise amino acid sequence for the purified protein (Fig. 7b). The spectrum showed a clearly visible peak at the position corresponding to the expected molecular mass of hirudin (Fig. 7c). After obtaining an expression yield of 6.68 mg/L culture, the purified protein concentration was found to be 1.67 mg/mL, using the Bradford method^31^.

### Antithrombin activity of hirudin

The antithrombin activity of purified hirudin and the commercially available product were 14,000 antithrombin units (ATU)/mL and 12,000 ATU/mL, respectively.

## Discussion

The present study describes the successful expression and production of recombinant hirudin from *H. nipponia.* The hirudin expression yield was found to be considerably higher than that reports for other hirudin variants or hirudin in leeches^26,32^. Recombinant hirudin was successfully obtained in the purified form, with the antithrombin activity comparable to commercially available hirudin.

Hirudin is currently the strongest known natural thrombin inhibitor, which interacts with thrombin to form a tight equimolar complex^33–36^. The structure of a protein determines its physicochemical properties and biological functions, and disulfide bonds play a critical role in protein stability by cross-linking different regions of polypeptide chains^37^. The three-dimensional structure of hirudin shows a tightly folded N-terminal globular region formed by three disulfide bonds and a flexible C-terminal tail^38^. Our analysis revealed that the putative hirudin protein was 6.97 kDa with six cysteine residues, which presumably form three pairs of disulfide bonds, and thus, might greatly strengthen and stabilize the tertiary structure of the mature protein^39^. In addition, multiple sequence alignment confirmed the presence of a highly conserved “core” motif (^34^CLC^36^) in the *H. nipponia* sequence, which is associated with hirudin’s binding to the active center of thrombin to block its hydrolytic activity^40^. Natural hirudin loses its anticoagulant properties on degradation or oxidation of the disulfide bonds^38^.

The methylotrophic yeast *P. pastoris* has been widely used, particularly after the release of its genome sequence in 2009, for producing various heterologous proteins^41^. More than 500 proteins have been successfully expressed in *P. pastoris* to date, including a variety of proteins with high medicinal or economic value, such as antibodies and vaccines^42^. High secretory capacity, a strong AOX promoter, and the presence of a glycosylation pathway are three distinct advantages of yeast expression systems over other eukaryotic systems^43–45^. In this study, the pPIC9K-hirudin recombinant plasmid was successfully constructed and effectively expressed in *P. pastoris* GS115. In addition, the target protein was secreted extracellularly, which further aided protein purification. However, the size of the target protein was not consistent with the putative molecular weight of hirudin, as determined using SDS-PAGE. The factors influencing exogenous gene expression in *P. pastoris* are complex and diverse, such as the structural, physical, and chemical characteristics of the exogenous protein; transformant phenotype; and the induction conditions^30^. Owing to the structural characteristics of hirudin, which predominantly exists as dimers or polymers, a multimeric aggregate appears on analysis using SDS-PAGE^46–48^. Thus, dimer formation may account for the molecular weight of hirudin protein observed in the present study, which was considerably higher than the expected molecular weight.

The thrombin titration method is commonly used to determine the specific activity of recombinant hirudin^6,9,10^. Several complex and varied factors, including the temperature, incubation time, and sample processing methods^6,15,29^, have been identified during the assay to determine antithrombin activity. Our results showed that the antithrombin activity of purified hirudin was 14,000 ATU/mL, which was higher than that obtained for commercially available hirudin products.

## Conclusions

In conclusion, a candidate hirudin gene was amplified from the salivary gland transcriptome of *H. nipponia*, and the resulting predicted amino acid sequence was characterized. Furthermore, a eukaryotic hirudin expression plasmid, *P. pastoris* GS115/pPIC9K-hirudin, was successfully constructed and characterized using SDS-PAGE and western blotting. Mass spectrometry analysis further confirm successful protein expression. The recombinant protein was expressed with a yield of 6.68 mg/L culture. The concentration and antithrombin activity of the purified hirudin was 1.67 mg/mL and 14,000 ATU/mL, respectively. These results lay the foundation for future studies to evaluate the structure and properties of hirudin, the development of anticoagulant drugs, and the large-scale production of purified, mature hirudin protein for commercial and medicinal applications.

## Methods

### Enzymes, vectors, media, and strains

T4 DNA ligase, KOD-Plus-Neo polymerase, EcoRI, NotI, and SacI were purchased from Bao Bioengineering, Co., Ltd. (Dalian, China). PMD18-T vector, pPIC9K vector, and *E. coli* DH5α were purchased from Shanghai Sangon Biotech Co., Ltd. (Shanghai, China). Minimal dextrose (MD), buffered glycerol-complex (BMGY), and buffered methanol-complex (BMMY) medium were prepared according to the recipe provided in the Invitrogen *Pichia pastoris* expression kit instruction manual.

### Animals and salivary tissue sample collection

All leeches were obtained from an adult *H. nipponia* colony grown in a medical leech breeding base of the Chongqing Academy of Chinese Materia Medica (Chongqing, China). Fifty healthy leeches were maintained in an aquaculture net cage filled with 15 L of dechlorinated tap water at 20–22 °C with a 12 h light/dark cycle prior to dissection. Every 5 days, half of the water was replaced with fresh water. Salivary tissue masses lying posterior to the three muscular jaws were removed aseptically using a sterilized dissecting tool; subsequently, they were rinsed in 0.5% bleach for 1 min followed by rinsing in deionized water for 1 min^49–51^. The tissues were then stored in RNAlater at − 80 °C (Qiagen, Hilden, Germany), according to the manufacturer’s specifications.

### RNA extraction and cDNA preparation

Total RNA was extracted from the aforementioned salivary tissues of *H. nipponia* using Trizol reagent (Tiangen, Beijing, China), according to the manufacturer’s instructions. RNA quality was assessed by electrophoresing on a 1.0% agarose gel, and the RNA concentration was determined using a NanoDrop 2000 spectrophotometer (Thermo Fisher Scientific, Waltham, MA, USA). First-strand cDNA was synthesized using the RevertAid First Strand cDNA Synthesis Kit (Thermo Fisher Scientific, Waltham, MA, USA).

### Molecular cloning of the hirudin gene of *H. nipponia*

Our previously isolated transcript (c16237_g1) from the salivary gland transcriptome databases of *H. nipponia*^27^ was screened for further analysis. Since this transcript showed the highest homology with the hirudin variant HV1(VV) (ALA22934.1), with an e-value of 3e^−13^, we designated this as a hirudin transcript. Amplification primers were designed using Primer Premier 5 software to clone the coding sequence of hirudin and were synthesized by Shanghai Sangon Co., Ltd. (Table 1). The cycling parameters were 94 °C for 2 min, 35 cycles of 94 °C for 30 s, 55 °C for 30 s, and 72 °C for 30 s, and final extension at 72 °C for 10 min. To obtain full-length cDNA of the hirudin gene, rapid amplification of 5′ and 3′ cDNA ends (RACE) was performed using a FirstChoice RLM-RACE Kit (Invitrogen, Waltham, MA, USA), according to the manufacturer’s instructions. Pairs of gene-specific primers were designed for 5′ and 3′ RACE PCR based on the acquired coding DNA sequence (listed in Table 1).

**Table 1:** Primers used in the present study.

Primer name	Sequence (5′–3′)	Application
Hir-F	GATCTGAAAAAATCTCAACG	CDS cloning
Hir-R	TTAATAATAATCGTAATCAG	
5′-RACE-1 (GSP1)	TCGGATTGCTGTCTGA	5′-RACE
5′-RACE-2 (GSP2)	ATGCTGAGCTTGAGACGT	
5′-RACE-3 (GSP3)	TGCCAACAGAACGACGAA	
3′-RACE-1	TTTGCATCTGCACGTCTCAAGCTCA	3'-RACE
3′-RACE-2	GGGCCCACCAAAGAAATGCATCA	
Hir-p-F	CTGAAGCTTACGTAGAATTCCAGCATTTCAAAGATTGC	Vector construction
Hir-p-R	GTCTAAGGCGAATTAATTCGCGGCCGCTTA**GTGGTGGTGGTGGTGGTG**ATAATAATC	
5′*AOX1*	GACTGGTTCCAATTGACAAGC	Identification of positive clones
3′*AOX1*	GCAAATGGCATTCTGACATCC	

EcoRI (GAATTC) and NotI (GCGGCCGC) are underlined. The six-histidine tag sequence is in bold.

*CDS* coding sequence, *RACE* rapid amplification of cDNA ends.

The PCR products were analyzed on 1.0% agarose gels, and the bands of interest were excised and purified with MiniBEST Agarose Gel DNA Extraction Kit (Takara Bio Inc., Shiga, Japan). The fragments were subcloned into a pMD-18 T vector and sequenced by Shanghai Sangon Co., Ltd.

### Sequence analysis and multiple sequence alignment of hirudin

Based on the cloned sequence, the full-length amino acid sequence of the hirudin protein was predicted using ORFfinder (http://www.ncbi.nlm.nih.gov/orffinder/). The molecular weight and theoretical pI were predicted using ProtParam (https://www.expasy.org/tools/protparam.html). Protein signal peptides were predicted using the SignalP 6.0 server (https://services.healthtech.dtu.dk/service.php?SignalP). The physicochemical properties of the hirudin protein after cleaving the signal peptides were predicted using the ExPASy ProtParam tool (https://web.expasy.org/protparam/).

A protein BLAST (http://www.ncbi.nlm.nih.gov/BLAST) search was performed based on the hirudin amino acid sequence. The other 17 sequences of hirudin from different leech species were obtained from the NCBI website and their relevant information is presented in Table 2. Multiple sequence alignment was accomplished using Clustal W ver. 2.0.10 and GeneDoc ver. 2.7.0.

**Table 2:** Relevant species and their hirudin proteins.

Species	Protein	Protein accession number	Identity (%)*	E-value	References**
*Hirudo nipponia*	Hirudin-HN	QDZ37419.1	57	6e−21	^26^
*Hirudo medicinalis*	HV2	ALA14576.1	53	2e−22	^52^
*Hirudo medicinalis*	HV3-PA	ALA22935.1	52	2e−23	^52^
*Hirudo orientalis*	HV3-PAF	APA20857.1	50	1e−22	^53^
*Hirudo medicinalis*	HV3-PA Subtype AFN	ALA22954.1	50	2e−22	^52^
*Hirudo medicinalis*	HV1(VV)	ALA22934.1	50	4e−24	^52^
*Hirudo verbana*	HV1-VV	APA20831.1	49	2e−22	^53^
*Hirudinaria manillensis*	HM1	Q07558.1	49	2e−22	^24^
*Hirudinaria manillensis*	HM2	P81492.2	48	9e−21	^24^
*Poecilobdella manillensis*	Hirudin	CAA51293.1	48	9e−21	^24^
*Poecilobdella manillensis*	HM3	APA20847.1	46	5e−16	^53^
*Poecilobdella viridis*	Hirudin	P84590	36	1e−09	^50^
*Hirudo medicinalis*	Hirudin	P28504	36	2e−09	^54^
*Hirudinaria manillensis*	Hirudin	P26631	33	1e−04	^55^
*Hirudinaria manillensis*	Hirudin P18	AAB21616.1	33	1e−04	^23^
*Hirudinaria manillensis*	Hirudin HV1	A53883	33	1e−07	^56^
*Hirudinaria manillensis*	Hirudin P6	AAB21615.1	30	3e−06	^23^

*Identity with hirudin was calculated using protein BLAST (protein vs. protein).

**The initial report of the hirudin sequence of these organisms.

### Construction of pPIC9K-hirudin recombinant plasmid

**Figure 8:**
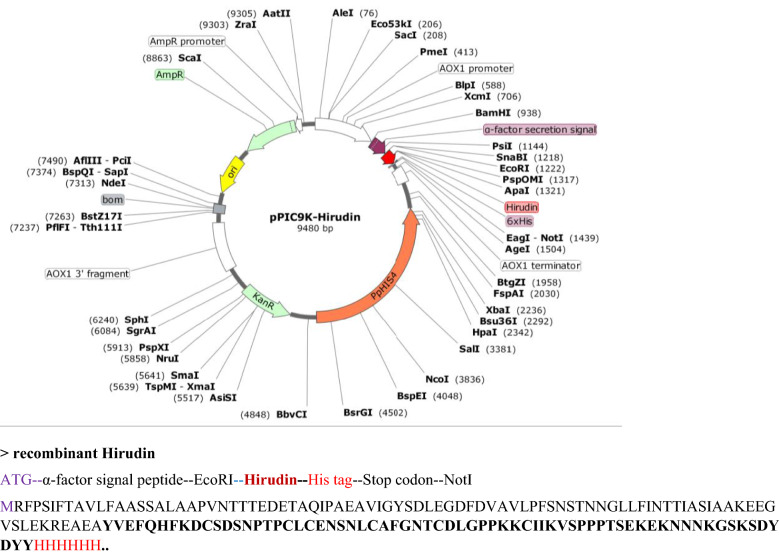
A schematic map of the recombinant plasmid pPIC9K-hirudin. (The target gene is marked in red).

Based on the gene sequencing results, codon optimization for eukaryotic expression was performed after cleaving the signal peptide. The sequence was amplified using appropriate primer pairs (Hir-p-F/Hir-p-R; Table 1) and KOD-Plus-Neo polymerase. Thus, appropriate restriction sites for the enzymes EcoRI (GAATTC) and NotI (GCGGCCGC) as well as protecting bases were added to 5′ and 3′ ends, respectively. In addition, a six-histidine tag was added to the C terminus of the hirudin gene. A schematic map of the constructed plasmid was designed using SnapGene Viewer ver. 4.0.4 based on the pPIC9K vector and the ORF of hirudin (Fig. 8). The pPIC9K vector and the PCR product (molar ratio of vector:insert was 1:3) were subjected to 2 h of double enzyme digestion using the same EcoRI and NotI enzymes at 37 °C and ligated with T4 DNA ligase. Competent *E. coli* DH5α cells were then transformed with recombinant plasmid pPIC9K-hirudin. The positive transformants were screened and verified using colony PCR and double enzyme digestion, and the PCR product was analyzed on a 1.5% agarose gel.

### Electro-transformation of *P. pastoris* GS115

*Pichia pastoris* GS115 competent cells were prepared according to the manual of Invitrogen Corporation. The recombinant expression plasmid was linearized using the SacI restriction enzyme and *P. pastoris* GS115 cells were transformed with the plasmid using electroporation. The resulting transformed cells were inoculated into 200 μL of MD medium and cultivated for 3 days at 30 °C to screen positive colonies. Single yeast colonies were retrieved from the MD plate using sterile toothpicks. Colony identification was confirmed using the PCR primers 5′ *AOX1* and 3′ *AOX1* (Table 1). PCR products were analyzed on a 1% agarose gel, and the target bands were excised and purified using MiniBEST Agarose Gel DNA Extraction Kit (Takara, Dalian, China). Positive clones were sent to Shanghai Bioengineering Technology Co., Ltd. for sequencing and further verification. *P. pastoris* GS115 transformants harboring pPIC9K-hirudin were designated as GS115/pPIC9K-hirudin, and *P. pastoris* cells transformed with the empty pPIC9K plasmid were used as the control and named GS115/pPIC9K.

### Induction and expression of recombinant yeast transformants 

The yeast clones expressing pPIC9K-hirudin were inoculated into a 250-mL bottle containing 50 mL BMGY medium overnight, after identification using PCR. The yeast was cultured at 28 °C with shaking at 250 rpm until the optical density at 600 nm (A_600_) reached 2–4. The yeast was then collected and induced in 80 mL of BMMY medium until the A_600_ reached 1.5 and cultured continuously at 28  °C for 3 days. During induction, the samples were collected every 24 h and methanol was added to obtain a final concentration of 1% (v/v). Samples were centrifuged at 12,000×*g* for 2 min at 4  °C. The supernatant and precipitate were collected and analyzed using a 15% SDS-PAGE, as described previously^57^.

### Western blot analysis

Supernatants and precipitates from GS115/pPIC9K-hirudin were loaded on a 150 g/L SDS-PAGE for protein separation and transferred to a polyvinylidene difluoride membrane through electrotransfer (54 mA, 80 min). After blocking with 1.5% bovine serum albumin (BSA) in blocking buffer at 4  °C overnight, the membrane was incubated with 1000 × dilutions of mouse-anti-His mAb (GenScript, Nanjing, China) at room temperature for 1 h, washed thrice with tris-buffered saline, and mixed with 1000 × dilutions of rabbit anti-mouse IgG antibody coupled with horseradish peroxidase (Invitrogen) at room temperature for 1 h. After washing, the membrane was treated with TrueBlue™ Peroxidase Substrate (Takara, Dalian, China) for 1 min.

### Purification of recombinant hirudin

The yeast clone expressing pPIC9K-hirudin was amplified, cultivated, and induced as described, and the culture supernatant was harvested for purification at 72 h post induction. The supernatant was carefully resuspended in a binding buffer (50 mM Tris–HCl, 150 mM NaCl, pH 8.0), loaded onto Ni-nitrilotriacetic acid resin (GenScript), washed, and eluted as recommended by the manufacturer. The protein purification system used an imidazole gradient (0–500 mM) as the target protein eluent. The samples were then analyzed using 15% SDS-PAGE and LC–MS/MS. The Bradford method^31^ was used to determine the concentration of the purified protein.

### Mass spectrometric analyses of purified hirudin

The purified protein was filtered through a 0.5-mL Ultracel-10K filter, and 5 µg protein was loaded on the BioAccord LC–MS System. The purified protein was first separated on an ACQUITY UPLC protein BEH C4 column and eluted from the column using gradient elution from 95% water with 0.1% formic acid to 95% acetonitrile with 0.1% formic acid. Intact protein was analyzed using a time-of-flight mass spectrometer with an electrospray ion source. Protein mass was deconvoluted using the UNIFI software suite.

### Antithrombin activity analysis

The antithrombin activity of purified recombinant hirudin was evaluated according to the Pharmacopoeia of the People’s Republic of China (2020 edition)^7^ and a previously reported method^8,15^. Commercially available hirudin (MedChem Express, Monmouth-Junction, USA) was used as the control. Briefly, antithrombin activity was quantitatively measured via titrating a solution of thrombin and expressed in ATU: one unit is defined as the neutralization of one NIH unit of thrombin (Sigma, Germany) at 37  °C. For the activity assay, 200 μL of 0.05% bovine fibrinogen solution prepared with 50 mM Tris–HCl buffer (pH 7.4) was thoroughly mixed with 100 μL of the sample, and then 5 μL of 40 NIH/mL thrombin solution (0.2 NIH unit) was added progressively and mixed gently. The above reaction mixture was incubated at 37 ± 0.5  °C for 1 min. The formation of a fibrin clot within 1 min was considered to be the endpoint of the titration. Otherwise, another 5 μL of thrombin solution was added continuously until a fibrin clot was observed.

### Ethical approval

We declare that the experiments described in this paper comply with the current laws in China. This article does not contain any studies with human participants performed by any of the authors.

## Supplementary Information

Supplementary Information.

## Data Availability

The datasets generated and/or analyzed during the current study are available in the “[NCBI] repository (https://www.ncbi.nlm.nih.gov/nuccore/MN116511)”.
