# Supplementary material for: Cloning, characterization, and heterologous expression of a candidate Hirudin gene from the salivary gland transcriptome of Hirudo nipponia
Source: Sci Rep. 2023 Mar 27;13:4943. doi: 10.1038/s41598-023-32303-2 (PMC10042815; doi:10.1038/s41598-023-32303-2)
Supplement: Supplementary file 1 — Supplementary Information. [file 41598_2023_32303_MOESM1_ESM.pdf]

Supplementary data

Title

Cloning, characterization, and heterologous expression of a candidate hirudin gene from the salivary gland transcriptome of *Hirudo nipponia*

Authors

Ping Shi[1,2,3,#], Jian Wei[4,#], Huajian You[1,2], Shijiang Chen[1,2], Fayin Tan[1,2], Zenghui Lu[1,2,3*]

Affiliations

1. Institute of Chinese Caterpillar Fungus, Chongqing Academy of Chinese Materia Medica, 34 Nanshan Road, 400065, Nan'an District, Chongqing, People's Republic of China.

2. Chongqing Sub-Center of National Resource Center for Chinese Materia Medica, China Academy of Chinese Medical Science, Chongqing 400065, People's Republic of China

3. Chongqing College of Traditional Chinese Medicine, Chongqing 402760, People's Republic of China

4. Department of TCM Geriatrics, Pucheng County Hospital, Shaanxi 715500, People's Republic of China

#These authors contributed equally to this work.

*To whom correspondence should be addressed:

Z.H. LU: E-mail: luzi520@yeah.net; Tel: 86-023-89029136; Fax: 86-023-89029087

Table of Contents

1 Supplementary Figures

1.1 Figures S1-S5

2 Supplementary sequence

2.1 Sequence S1

2.2 Sequence S2

1 Supplementary Figures

1.1 Figures S1-S5

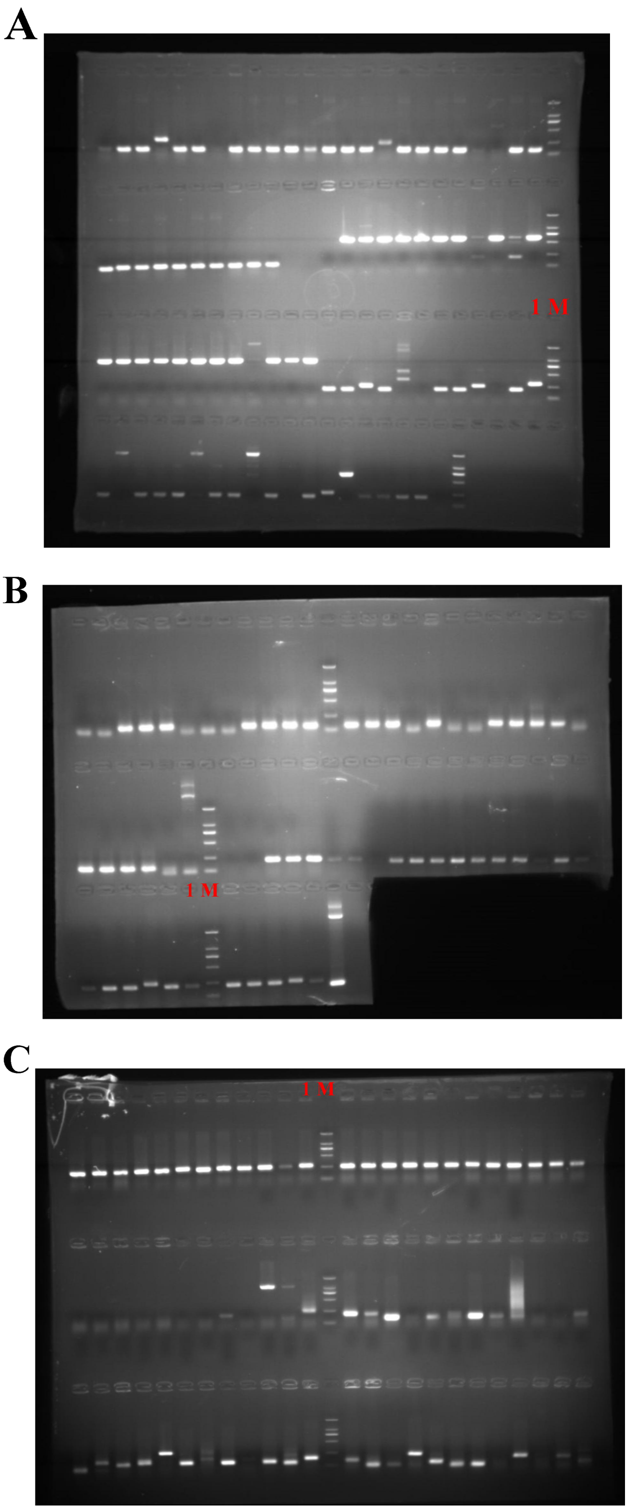

Figure S1. Uncropped gel image of Fig.1. (Lane 1 which marked in red is target fragment. Lane M which marked in red is DL2000 Marker. The rest of the bands were not relevant to this study.) A:

Intermediate fragment amplification. B: 5′-RACE amplification. C: 3′-RACE amplification. The PCR products were analyzed on a 1% agarose gel.

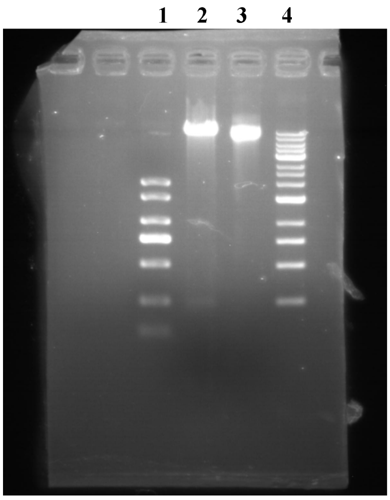

Figure S2. Uncropped gel image of Fig.4. Lane information: first lane is Marker DL 2000 plus. Lane 2 is recombinant plasmid digested with EcoRI and NotI, lane 3 is recombinant plasmid, and lane 4 is Marker DL 10000. The products were analyzed on a 1% agarose gel.

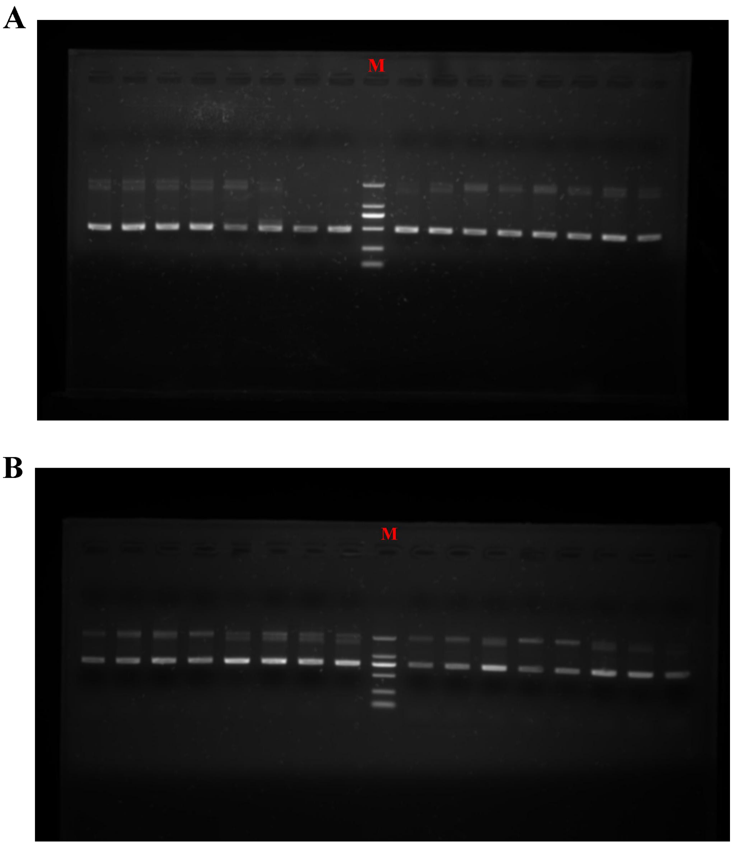

Figure S3. Uncropped gel image of Fig.5. (Lane M which marked in red is Marker DL2000. The rest of the bands were target products.) A: *P. pastoris* GS115 cells transformed with the empty pPIC9K plasmid (GS115/pPIC9K). B: *P. pastoris* GS115 transformants with pPIC9K-*Hirudin* (GS115/pPIC9K-*Hirudin*). The PCR products were analyzed on a 1.5% agarose gel.

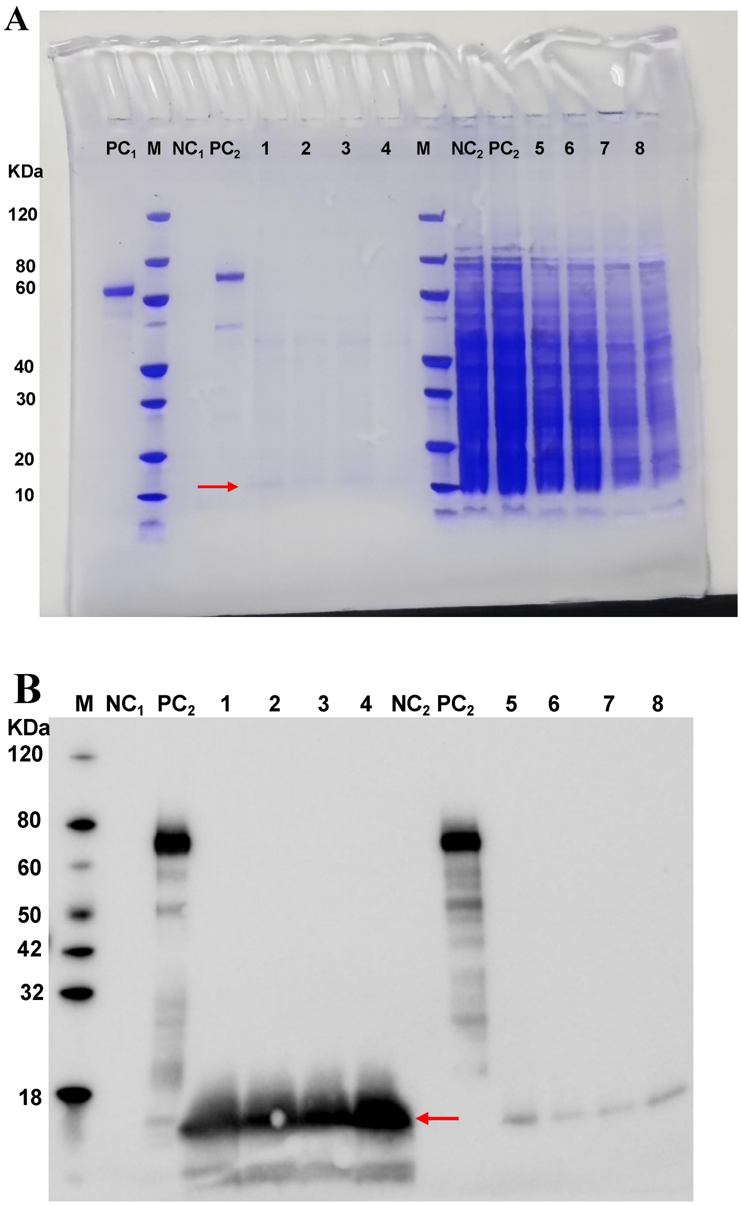

Figure S4. Uncropped gel image of Fig.6. (Lane M: Protein MW marker (Broad); Lane PC_1: BSA (1μg); Lane PC_2: Positive control (GenScript); Lane NC_1: Medium without induction; Lane NC_2: Cell pellet without induction. Lane 1: Supernatants from GS115/pPIC9K-*Hirudin* after 24 h induction; Lane 2: Supernatants from GS115/pPIC9K-*Hirudin* after 48 h induction; Lanes 3, 4: Supernatants from GS115/pPIC9K-*Hirudin* after 72 h induction; Lane 5: Precipitate from GS115/pPIC9K-*Hirudin* after

24 h induction; Lane 6: Precipitate from GS115/pPIC9K-*Hirudin* after 48 h induction; Lanes 7, 8: Precipitate from GS115/pPIC9K-*Hirudin* after 72 h induction.) A: SDS-PAGE analysis of pPIC9K-*Hirudin* expression in recombinant *P. Pastoris*. B: Western blot analysis of recombinant hirudin. The target protein is indicated with a red arrow (~15 kDa). The recombinant hirudin was analyzed on a 15% SDS-PAGE gel.

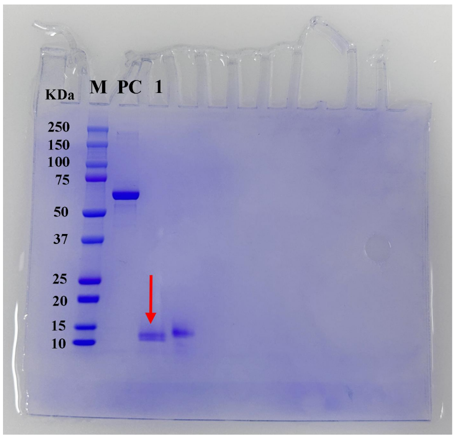

Figure S5. Uncropped gel image of Fig.7**a**. (Lane M: Protein marker; Lane PC: BSA (1μg); Lane 1: Purified hirudin. The rest of the band was not relevant to this study.) The target protein is indicated with a red arrow. The purified hiduin was analyzed on a 15% SDS-PAGE gel.

2 Supplementary sequence

2.1 Sequence S1

>c16237_g1

TGCATCATAAAAGTATCACCACACAGCCTTTCAGGATCTTCAATCGGATCTGAAAAAAT

CTCAACGATGTTCTCTCTGAAATTGTTCGTCGTTCTGTTGGCAGTTTGCATCTGCACGT

CTCAAGCTCAGCATTTCAAAGATTGCTCAGACAGCAATCCGACTCCATGCTTGTGCGA

AAATAGTAATCTCTGTGCTTTTGGTAACACTTGTGATCTGGGCCCACCAAAGAAATGCA

TCATAAAAGTATCACCACCTCCCACCTCGGAGAAAGAGAAAAATAACAACAAAGGAA

GTAAATCTGATTACGATTATTATTAA

2.2 Sequence S2

2.2.1 Sequencing chtomatogram

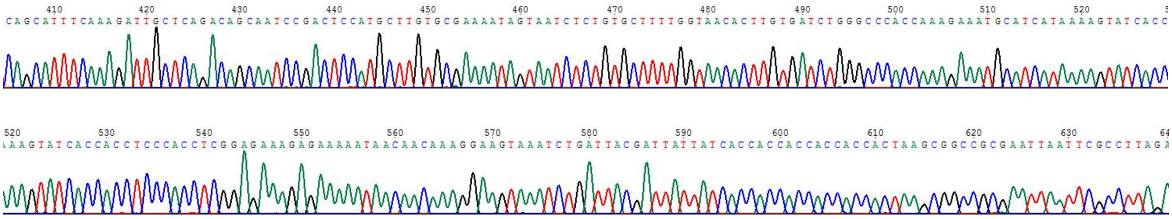

2.2.2 DNA base sequence

>positive clone

GCGTATACGACTTTACGACACTTGAGAAGATCAAAAAACAACTAATTATTCGAAGGATC

CAAACGATGAGATTTCCTTCAATTTTTACTGCAGTTTTATTCGCAGCATCCTCCGCATTA

GCTGCTCCAGTCAACACTACAACAGAAGATGAAACGGCACAAATTCCGGCTGAAGCT

GTCATCGGTTACTCAGATTTAGAAGGGGATTTCGATGTTGCTGTTTTGCCATTTTCCAAC

AGCACAAATAACGGGTTATTGTTTATAAATACTACTATTGCCAGCATTGCTGCTAAAGAA

GAAGGGGTATCTCTCGAGAAAAGAGAGGCTGAAGCTTACGTAGAATTCCAGCATTTCA

AAGATTGCTCAGACAGCAATCCGACTCCATGCTTGTGCGAAAATAGTAATCTCTGTGCT

TTTGGTAACACTTGTGATCTGGGCCCACCAAAGAAATGCATCATAAAAGTATCACCACC

TCCCACCTCGGAGAAAGAGAAAAATAACAACAAAGGAAGTAAATCTGATTACGATTAT

TATCACCACCACCACCACCACTAAGCGGCCGCGAATTAATTCGCCTTAGACATGACTGT

TCCTCAGTTCAAGTTGGGCACTTACGAGAAGACCGGTCTTGCTAGATTCTAATCAAGA

GGATGTCAGATCCCCATTTGGCC

Note: The sequence of *hirudin* is marked with a straight line.
